# Appearance of the bare area of the proximal radius on magnetic resonance imaging: preliminary assessment on its potential usefulness in preoperative planning for distal biceps brachii tendon repair

**DOI:** 10.1007/s00256-026-05175-6

**Published:** 2026-02-20

**Authors:** Michal Benes, Petr Fulin, Bruno Jurasek, Vladislav Bartak, David Kachlik, Vojtech Kunc

**Affiliations:** 1 1st Department of Orthopaedics, First Faculty of Medicine, Charles University and Motol University Hospital, Prague, Czech Republic; 2 Department of Anatomy, Second Faculty of Medicine, Charles University, Prague, Czech Republic; 3 Center for Endoscopic, Surgical and Clinical Anatomy (CESKA), Second Faculty of Medicine, Charles University, Prague, Czech Republic; 4 Department of Radiology, Second Faculty of Medicine, Charles University and Motol University Hospital, Prague, Czech Republic

**Keywords:** Bare area, Deep branch of radial nerve, Distal biceps brachii tendon injury, Magnetic resonance imaging, Posterior interosseous nerve, Supinator muscle, Variation

## Abstract

**Objective:**

Bare area of the proximal radius (BA) is a variable osseous component within the floor of the supinator canal, caused by a slip between the insertions of the two layers of the supinator muscle. As this variable structure potentially increases the likelihood of iatrogenic injury to the deep branch of the radial nerve (DBRN) during distal biceps brachii tendon repair, this study aimed to assess the appearance of the BA on MRI to evaluate its usefulness in preoperative planning for reinsertion procedures.

**Materials and methods:**

A total of 103 elbow MRIs of adult patients were retrospectively included in the study. Each elbow was evaluated in transverse, sagittal, and coronal planes by two observers. Unclear cases were resolved by a consensus among all authors. In case of the presence of the BA, its width was measured, its extent was compared with the level of the radial tuberosity, and its relationship with the DBRN was noted. Inter- and intraobserver agreement was assessed with the use of Cohen’s *κ* coefficient.

**Results:**

The BA was detected in 49.5% of cases, with a mean width of 4.6 mm. Extent of the BA frequently overlapped with the level of radial tuberosity in 70.6% of cases. The DBRN was in direct contact with the periosteum due to the presence of the BA in 29.4% of cases. No statistically significant differences were found between sides and sexes. Inter- and intraobserver reliability reached almost perfect agreement (all *κ* > 0.845).

**Conclusion:**

Given the frequent presence of the BA, MRI appears to be suitable for preoperative detection of the BA and assessment of its relationship with the DBRN. Preoperative MRI in distal biceps brachii tendon ruptures seems to be beneficial for surgical planning of the fixation device choice and placement.

## Introduction

Neurological deficits following distal biceps brachii tendon repair are among the most common complications. The spectrum of neural deficits ranges from mild sensory dysesthesia to severe motor palsy. The most frequent motor nerve injury, although appearing only in about 1.6% of cases, is the postoperative posterior interosseous nerve (PIN) palsy [1]. The mechanisms of injury include neural ischemia due to retractor placement, laceration resulting from aggressive dissection, direct drill bit overshoot injury or fixation device injury within the supinator canal, or delayed palsy caused by mechanical irritation of the deep branch of the radial nerve (DBRN) [1–3].

**Fig. 1:**
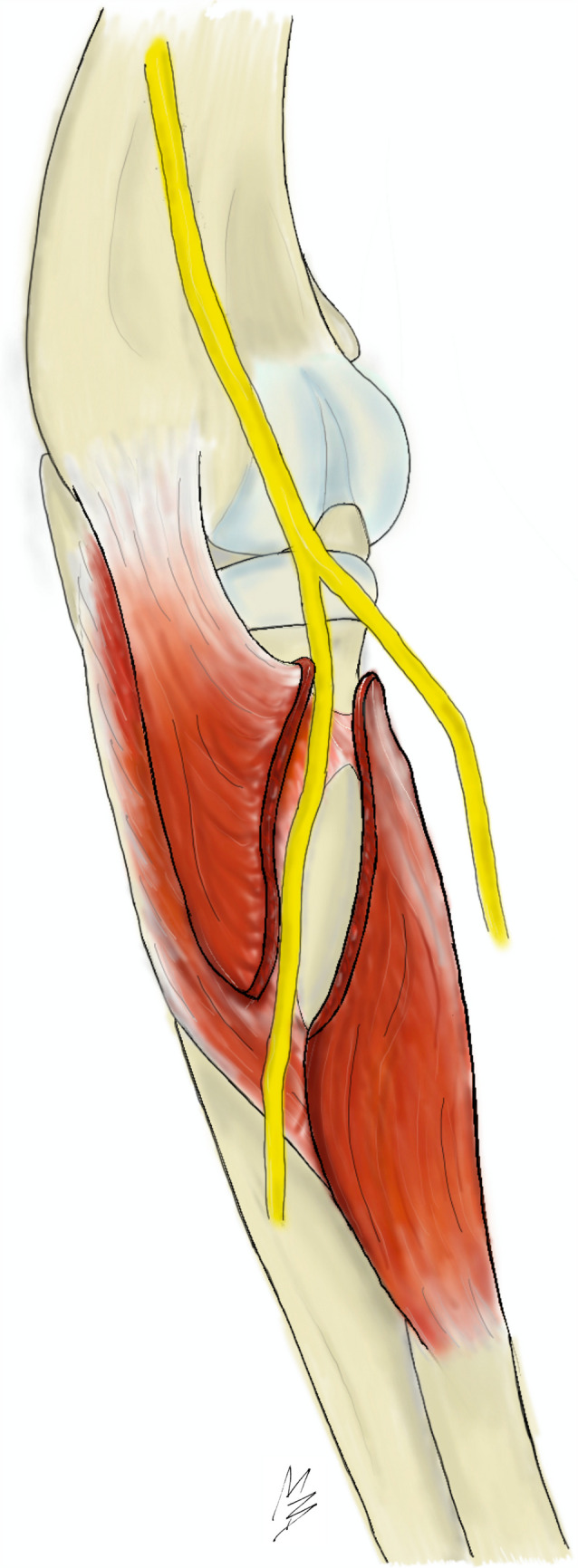
Schematic drawing of the bare area

Single-incision distal biceps brachii tendon repair is a preferable approach to the treatment of distal (insertional) biceps brachii tendon ruptures in many institutions. While using this technique, it is believed that the risk of iatrogenic injury may be preventable by respecting safe drilling angles or by using monocortical fixation devices. Recently, emphasis has been drawn to a variable osseous component within the supinator canal, termed the bare area of the proximal radius (BA) [2, 4]. This structure represents a gap between insertion sites of the two supinator muscle layers, exposing the periosteal surface to a direct contact with the DBRN coursing through the supinator canal (Fig. 1), thus increasing the likelihood of neural iatrogenic injury when drilling through the far cortex. Although optimal drilling trajectories for anatomical repair have been suggested to avoid DBRN injury [5–10], the desired drilling angles may not be feasible due to limited forearm movement which hinders sufficient exposure of the radial tuberosity.

Although physical examination and ultrasound are usually sufficient to diagnose distal biceps brachii tendon ruptures, magnetic resonance imaging (MRI) poses an additional imaging method of choice that maps the anatomy in more detail [11, 12]. The aim of this study was to evaluate the appearance of the BA on MRI to assess its utility in preoperative planning for choosing the appropriate technique and fixation device in distal biceps brachii tendon repair in order to decrease the likelihood of DBRN injury. We hypothesized that the BA would be clearly visible on MRI and thus could be potentially used in preoperative planning.

## Material and methods

### Sample and study selection

**Fig. 2:**
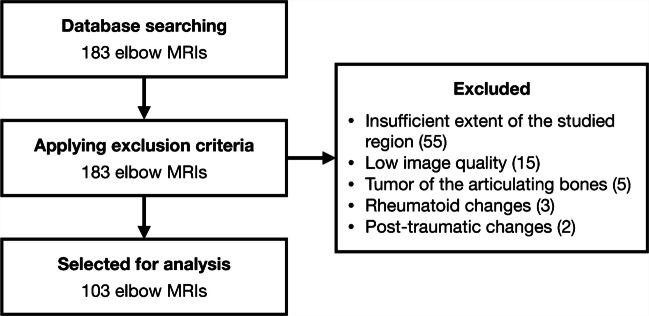
Flowchart of the MRI inclusion for analysis

Following institutional review board approval, the radiological database of the 1st Department of Orthopaedics, First Faculty of Medicine, Charles University and Motol University Hospital, Prague, Czech Republic was searched to compile all available elbow MRI scans performed between August 2018 and August 2024. The search yielded a total of 183 adult MRI studies of the elbow, which underwent screening for eligibility. The criteria for inclusion comprised MRI studies with high-quality images in all anatomical planes with at least 4-mm slice thickness. Studies were excluded if they (1) did not show the entire extent of the supinator canal; (2) had insufficient quality; (3) did not include all desired sequences and slices; (4) had severe degenerative, acute traumatic, or posttraumatic changes; and (5) the bones forming the elbow joint were affected by a tumor. After applying the exclusion criteria, 80 MRIs were withdrawn, while the remaining 103 MRIs fulfilled the criteria for inclusion in the study (Fig. 2).

### MRI evaluation

Elbow MRI examinations were performed using clinical scanners operating at 1.5 T and 3 T field strengths. All patients underwent a standardized imaging protocol that included T1-weighted and proton-density (PD) sequences acquired in sagittal, transverse, and coronal planes. Imaging parameters were adjusted according to magnet strength. Each patient’s MRI was evaluated in all available views using the MARIE PACS software (OR-CZ, Czech Republic). If the BA (representing an osseous gap between the muscular insertions of the deep and superficial layers of the supinator muscle) was noted, additional evaluation was carried out. The widest distance (considered as width) of the BA was measured in a transverse plane using the built-in distance measuring tool. Each measurement was performed twice, and an average value was then used for analysis. Proximo-distal extent of the BA was compared in regard to the level of the radial tuberosity. The DBRN was also identified, and its relationship with the BA was noted. The images were evaluated by two independent observers, including an orthopedic surgeon with 3 years of experience and a radiologist with 5 years of experience. Each MRI was evaluated twice within a 2-week period to assess the intraobserver and interobserver reliability. All disagreements and inconclusive findings were resolved by a consensual decision between the listed authors.

### Statistical analysis

Categorical variables are expressed as counts and percentages. Continuous variables are presented as means ± standard deviations. The *chi*-squared test was employed to compare the categorical variables. For continuous variables, differences were assessed using the Mann–Whitney *U* test. A *p* ≤ 0.05 was considered statistically significant. Inter- and intraobserver reliability was tested using Cohen’s *κ* coefficient. Values for *κ* were considered as 0–0.2 = poor agreement; 0.3–0.4 = fair agreement; 0.5–0.6 = moderate agreement; 0.7–0.8 = strong agreement; and > 0.8 = almost perfect agreement. All analyses were conducted using GraphPad Prism version 10.4.0 (GraphPad Software, USA).

## Results

Out of the 103 elbow MRI studies, there were 53 males and 50 females (51.5% vs. 48.5%) with a mean age of 39.7 ± 18.0 (range 18–94) years. Fifty-eight elbows were right and 45 were left (56.3% vs. 43.7%). The MRIs were indicated due to unspecified elbow pain, suspected but unconfirmed tumor mass, or elbow instability.

**Fig. 3:**
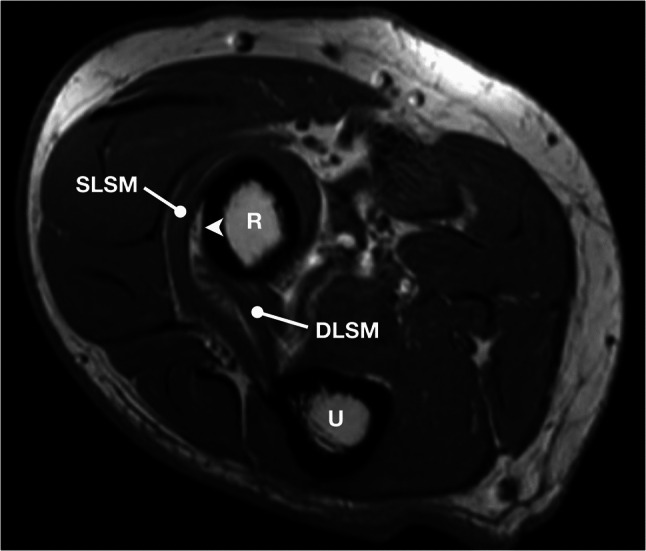
Right elbow MRI showing the bare area (arrowhead) with a direct contact with the deep branch of the radial nerve. DLSM = deep layer of the supinator muscle; R = radius; SLSM = superficial layer of the supinator muscle; U = ulna

**Fig. 4:**
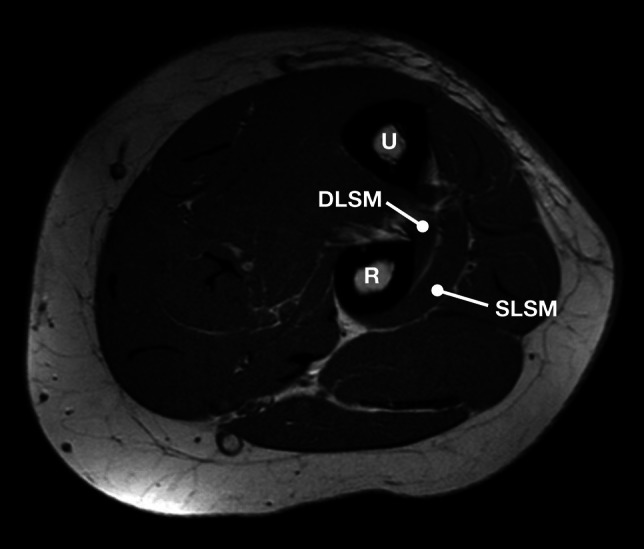
Left elbow MRI without the bare area. DLSM = deep layer of the supinator muscle; R = radius; SLSM = superficial layer of the supinator muscle; U = ulna

The BA was identified in 51 elbows (49.5%) (Fig. 3). There were no statistically significant differences in incidence between the sides and sexes (Table 1). The BA demonstrated a focal area along the posterolateral aspect of the proximal radius that appeared hyperintense on T1-weighted and PD non–fat-saturated sequences, with corresponding hypointensity on proton-density fat-saturated images. These signal characteristics are consistent with fat-containing connective tissue adherent to the adjacent osseous surface. The mean width of the BA was 4.6 ± 1.4 mm with no significant difference between sides and sexes (Table 1). In the remaining 52 elbows (50.5%), there was no BA detected (Fig. 4).

**Table 1:** Results of comparative analysis between sides and sexes

	Total (*n* = 103)	Right (*n* = 58)	Left (*n* = 45)	*p*	Male (*n* = 53)	Female (*n* = 50)	*p*
Presence of BA	51 (49.5%)	28 (48.3%)	23 (51.1%)	0.739	27 (50.9%)	24 (48.0%)	0.822
Width (mm)	4.6 ± 1.4	4.7 ± 1.6	4.4 ± 1.4	0.838	4.8 ± 1.8	4.5 ± 1.4	0.524
Relation to RT							
Within Below	36 (70.6%) 15 (29.4%)	19 (67.9%) 9 (32.1%)	17 (73.9%) 6 (26.1%)	0.272	20 (74.1%) 7 (25.9%)	10 (41.7%) 14 (58.3%)	0.094
Periosteal contact with DBRN	15 (29.4%)	10 (35.7%)	5 (21.7%)	0.248	8 (29.6%)	7 (29.2%)	0.999

The BA was always present on the posterolateral aspect of the proximal radius, opposite to the radial tuberosity. The extent of the BA overlapped with the extent of the radial tuberosity in 36 cases (70.6%), while in the remaining 15 cases (29.4%) the whole BA was located below the level of the radial tuberosity. The distal margin of the BA was always below the radial tuberosity. There were no statistically significant differences between sides and sexes when comparing the location of the BA (Table 1).

**Fig. 5:**
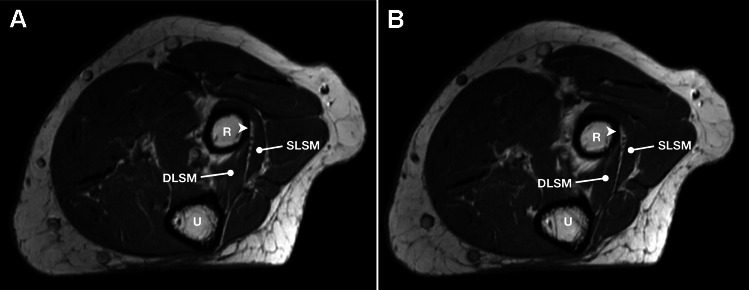
Right elbow MRIs showing the change of deep branch of the radial nerve location in regard to the bare area (arrowhead) when traveling from proximal (**A**) to distal (**B**) direction. Note the deep branch of the radial nerve approaching the bare area distally (**B**). DLSM = deep layer of the supinator muscle; R = radius; SLSM = superficial layer of the supinator muscle; U = ulna

The DBRN laid on the periosteum of the proximal radius in 15 cases (29.4%). No statistically significant differences were detected between sides and sexes (Table 1). There were no cases where the DBRN was in contact throughout the whole length of the BA. Instead, the DBRN travelled proximally over the deep layer of the supinator muscle and came in contact with the BA as it coursed distally toward the exit of the supinator canal (Fig. 5).

Inter- and intraobserver agreement for detection of the BA was almost perfect (Table 2).

**Table 2:** Results of inter- and intraobserver reliability. 95% CI = 95% confidence interval

	*κ*	95% CI
Interobserver agreement	0.845	0.741–0.948
Intraobserver agreement		
Observer 1 Observer 2	0.903 0.961	0.820–0.986 0.908–1.000

## Discussion

Although the presence of the BA appears to be a risk factor for DBRN injury during distal biceps brachii tendon repair, the BA is rarely mentioned in the contemporary literature. Therefore, the current study aimed to provide an assessment of the BA on MRI and its potential utility in preoperative planning for distal biceps brachii tendon repair. Our findings demonstrate that the BA was present in half of the elbow MRI scans. Moreover, a significant number of cases exhibiting the BA featured direct contact between the DBRN and the periosteum of the proximal radius. These findings highlight the relevance to surgical safety when performing distal biceps brachii tendon repair, advocating for more cautious preoperative planning and a resultant personalized treatment approach.

Previous anatomical investigations reported variable incidences of the BA. The first known description by Davies and Laird noted its presence in 57% of 21 specimens [4]. Lawton et al. observed its occurrence in 71% out of 24 specimens [13]. Moreover, Gilan et al. documented a markedly lower incidence of 10% among 40 upper limbs [14]. Most recently, Benes et al. identified the BA in 56.0% of 100 upper limbs [15]. Our study on 103 elbows of Central-European population found the BA in 49.5% of cases, which falls within the range detected in earlier reports. However, to date, there has been no description of the BA on MRI; thus, our data are only comparable to the above-listed cadaveric studies. Nonetheless, the similarity in incidence rates between previous cadaveric studies and ours suggests that MRI recognizes the BA and appears suitable for preoperative assessment prior to reinsertion procedures. Furthermore, the slight discrepancies in incidence among different studies may be attributable to differences in sample sizes, population demographics, and applied methodology.

The identification of the BA is particularly pertinent to the planning of bicortical drilling trajectories for distal biceps brachii tendon repair. Historically, the literature has offered various recommendations regarding optimal drilling angles, yet none of the studies considered the possible presence of the BA. For instance, Bain et al. advocated for perpendicular anterior-to-posterior drilling, cautioning against distal and radial directions [5]. Conversely, others have suggested 0–30° ulnar angulations, with some evidence indicating that angulations around 30° in the ulnar direction provide safer distances from the DBRN [6–8]. Based on our results, the DBRN is especially at risk of injury when drilling is directed distally as it frequently lies immediately on the periosteum of the proximal radius (in the presence of the BA). These findings align with recommendations of Duncan et al. and Becker et al. who suggested more proximally oriented drilling [9, 10]. An intriguing aspect of our results is the frequent overlap between the extent of the BA and the radial tuberosity in approximately 71% of cases, with the remaining 29% exhibiting a BA limited below the radial tuberosity level. These findings further support the need for individualized preoperative planning, as the BA’s location relative to the radial tuberosity could influence surgical approach and fixation strategies.

**Fig. 6:**
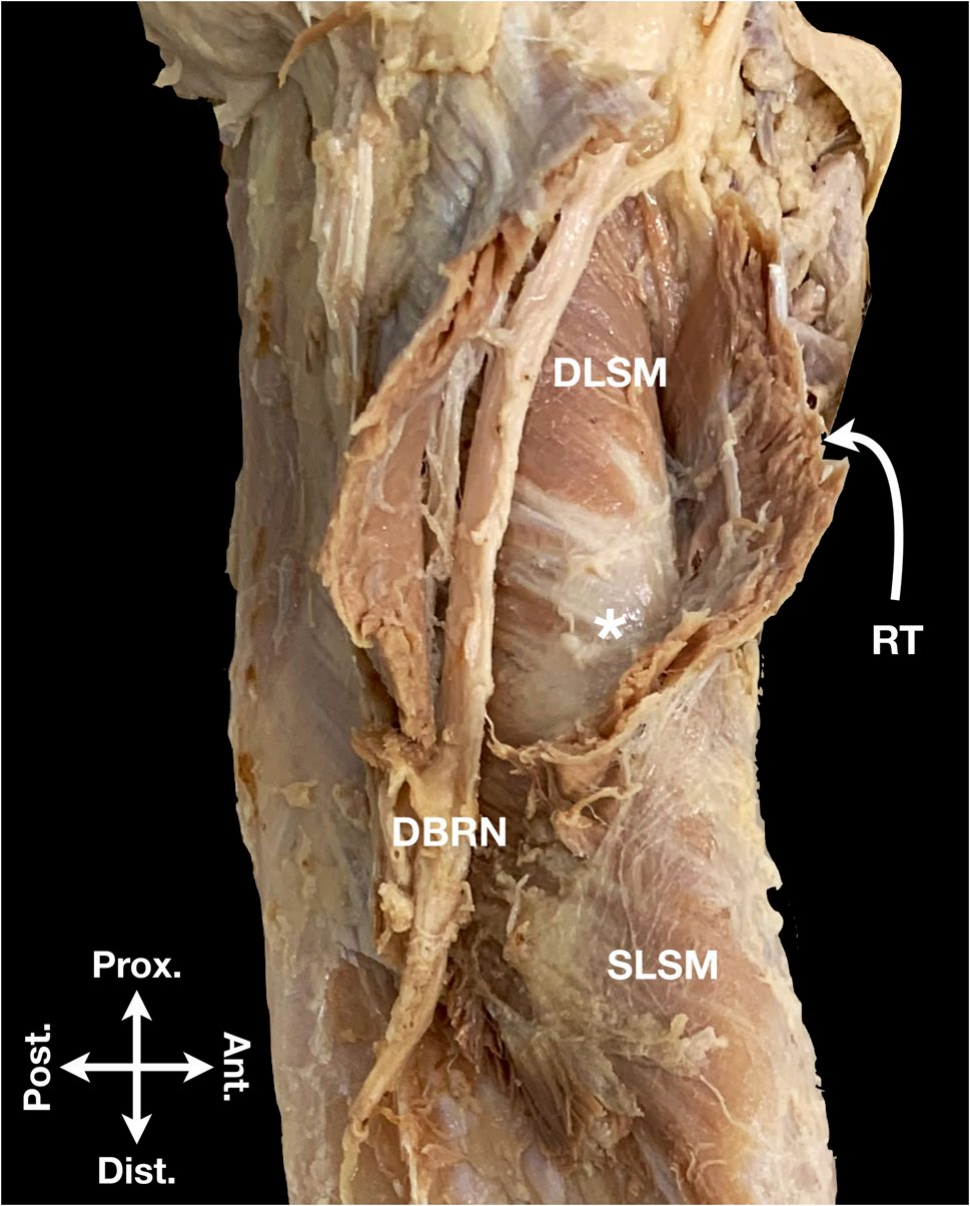
Anatomical specimen with incised superficial layer of the supinator muscle (SLSM) demonstrating the bare area (asterisk) and course of the deep branch of the radial nerve (DBRN). Ant. = anterior; Dist. = distal; DLSM = deep layer of the supinator muscle; Post. = posterior; Prox. = proximal; RT = radial tuberosity

The periosteal contact of the DBRN within the supinator canal was reported with incidences ranging from 2% to over 80% [4, 13–17]. Our study observed the periosteal contact of the DBRN with the BA in 29.4% of cases, but it never maintained contact throughout the entire BA length. Instead, the nerve proximally traversed over the deep layer of the supinator muscle, approaching the BA distally (Fig. 6). Notably, as the DBRN is not static, it exhibits proximal movement during pronation and distal movement during supination [18]. These dynamic shifts imply that hardware protruding above the cortex within the BA could cause intermittent or chronic nerve irritation, especially as the nerve moves relative to fixed implants. Such irritation could manifest clinically as acute as well as delayed PIN palsy.

Neural complications following distal biceps brachii tendon repair are common. Although sensory nerves are most frequently involved, PIN palsy (DBRN changes its name to PIN after it leaves the supinator canal [19]) stands as a major complication with potentially devastating consequences [1]. To reduce the risk of PIN injury, surgical strategies should aim to avoid perforating the far cortex from the radial tuberosity, especially in cases where the BA is present. Techniques such as unicortical fixation or precise angulation of drilling to stay clear of the BA may optimize functional outcomes and minimize nerve-related complications [2, 20]. Besides the relevance for distal biceps brachii tendon repair, the intimate relationship of the DBRN to the periosteum of the proximal radius increases the risk of its damage in case of fractures at this site [21].

This study has several limitations. Position of the upper limb was not constant among the performed MRIs as the individual’s range of motion, individual habitus, and positioning by the operating assistant differed from case to case. Therefore, the relationship between the DBRN and the BA should be considered with caution due to the dynamic nature of the DBRN during forearm movement. Furthermore, we did not assess any real-time movement of the DBRN within the supinator canal to obtain data regarding safe upper limb positioning during the reconstructive procedures. Only MRIs of adult patients were enrolled in this study because the primary goal was to implicate our findings for the repair of distal biceps brachii tendon ruptures, which predominantly occur in active adults. Studying MRIs of the whole age spectrum in future studies could provide insight into the development of the BA. Although effort was made to minimize the risk of bias when evaluating the MRIs, consensus bias in unclear cases cannot be ruled out. Moreover, a significant number of MRI studies did not meet the inclusion criteria due to insufficient quality or extent of the imaging site, which must be considered in clinical practice, and attention must be paid to performing high-quality images. Despite these limitations, we introduce the first study implementing MRI in the detection of the BA in order to report on its usefulness in preoperative planning. However, a future diagnostic accuracy study should be performed to verify the MRI performance as the current study is rather a morphological assessment.

In conclusion, the BA is a frequent variation within the supinator canal, exposing the periosteal surface to a contact with the DBRN. This topographical arrangement potentially increases the likelihood of neural injury during reinsertion procedures for ruptured distal biceps brachii tendon. Given the similar incidences among anatomical literature and our radiological findings, preoperative elbow MRI appears to reliably display the BA and should be considered when performing the single-incision distal biceps brachii tendon repair to plan an optimal reconstructive strategy and decrease the risk of DBRN injury.

## Data Availability

Data sharing inquiries can be made by request to the corresponding author.
